# Nausea, Vertical Gaze Palsy, and Excessive Sleep: An Unusual Presentation of Pediatric AQP4-Antibody Positive Neuromyelitis Optica Spectrum Disorder

**DOI:** 10.1055/a-2625-0994

**Published:** 2025-06-11

**Authors:** Tatjana A. Oberholzer, Leonie Plastina, David Wille, Selma Sirin, Annette Hackenberg

**Affiliations:** 1 Department of Pediatric Neurology, University Children's Hospital, Zürich, Switzerland; 2 Department of Diagnostic Imaging, University Children's Hospital, Zürich, Switzerland

**Keywords:** vertical gaze palsy, narcolepsy, NMOSD, pediatric onset, AQP4-antibody

## Abstract

Neuromyelitis optica spectrum disorder (NMOSD) is a rare neuroinflammatory disease with an annual incidence of less than 1 case in 1,000,000 in the White population and a median age of onset at 40 years. NMOSD usually presents with optic neuritis and longitudinally extensive transverse myelitis. Various brainstem, cerebellar, diencephalic, and hemispheric symptoms may also occur. Early diagnosis and treatment are crucial for symptom management and prevention of relapses and disability. We report the case of a prepubertal girl, highlighting unique clinical and magnetic resonance imaging features and the risk of early parenchymal damage.

## Case Presentation

An 11-year-old Caucasian female without any medical history was presented to the emergency room with a 10-day history of nausea, vomiting, and progressive fatigue. There were no identified trigger factors such as previous infections or stress, and no chronic diseases were reported within the family. Excessive sleep evolved, with very short periods of wakefulness (a few minutes), during which she was orientated and able to solve simple tasks but complained of tiredness. Nutritional support via gastric tube was initiated.

Three days after admission she developed bilateral vertical gaze palsy and absent pupillary responses. A comprehensive ophthalmological evaluation could not be performed due to sleepiness. Small objects could be recognized and named; fundoscopy was unremarkable. These findings led to the suspicion of a mesencephalic lesion involving the pretectal area/third nerve nucleus.

**Fig. 1:**
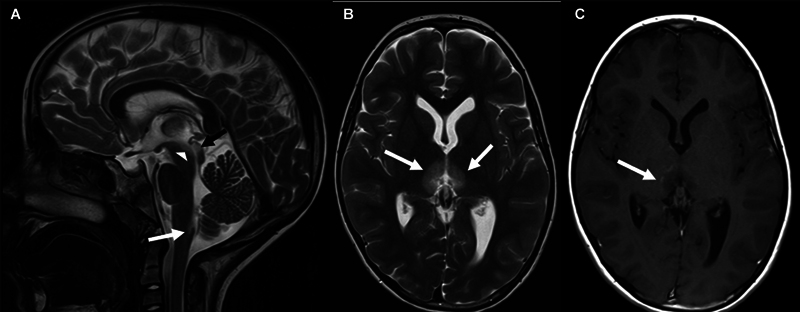
Initial brain magnetic resonance imaging ( **A/B** : T2-weighted sequences sagittal/transversal, ( **C** ) T1-weighted sequence transversal after contrast administration) of the 11-year-old girl showing edema in the area postrema ( *white arrow* in a), the periaqueductal gray/pretectal area ( *white arrowhead* in **A** ), the superior colliculi ( *black arrow* in **A** ) and the medial thalami ( *white arrows* in **B** ). A slight enhancement is seen in the periphery after gadolinium administration ( *white arrow* in **C** ).

Cranial magnetic resonance imaging (MRI) revealed symmetrical T2-/FLAIR-hyperintense edema in both medial thalami extending to the periependymal surface of the third ventricle and into the mesencephalon, involving the periaqueductal gray, pretectal area, and the superior colliculi of the quadrigeminal plate ( Fig. 1 ). Another area of edema was observed in the area postrema of the medulla oblongata. The lesions did not cause any mass effect, showed slight contrast enhancement at the periphery, no diffusion restriction, and no hemorrhage. No pathological findings were found in the supratentorial white matter, the optic nerves, the optic chiasm, or the spinal cord.

Differential diagnosis included inflammatory and metabolic disorders. Cerebrospinal fluid (CSF) analysis revealed mononuclear pleocytosis (29/µL), normal lactate, protein levels, and borderline glucose (2.2 mmol/L). Oligoclonal bands (OCB) were negative. Anti-Aquaporin-4-IgG antibodies (AQP4) were positive in both CSF (1:32) and serum (1:320), whereas anti-MOG IgG antibodies and various neuronal antibodies were negative. Orexin in CSF was 172 pg/mL, below the normal range (>200 pg/mL). Thiamine in CSF and amino acid profiles in blood and CSF revealed no abnormalities.

**Fig. 2:**
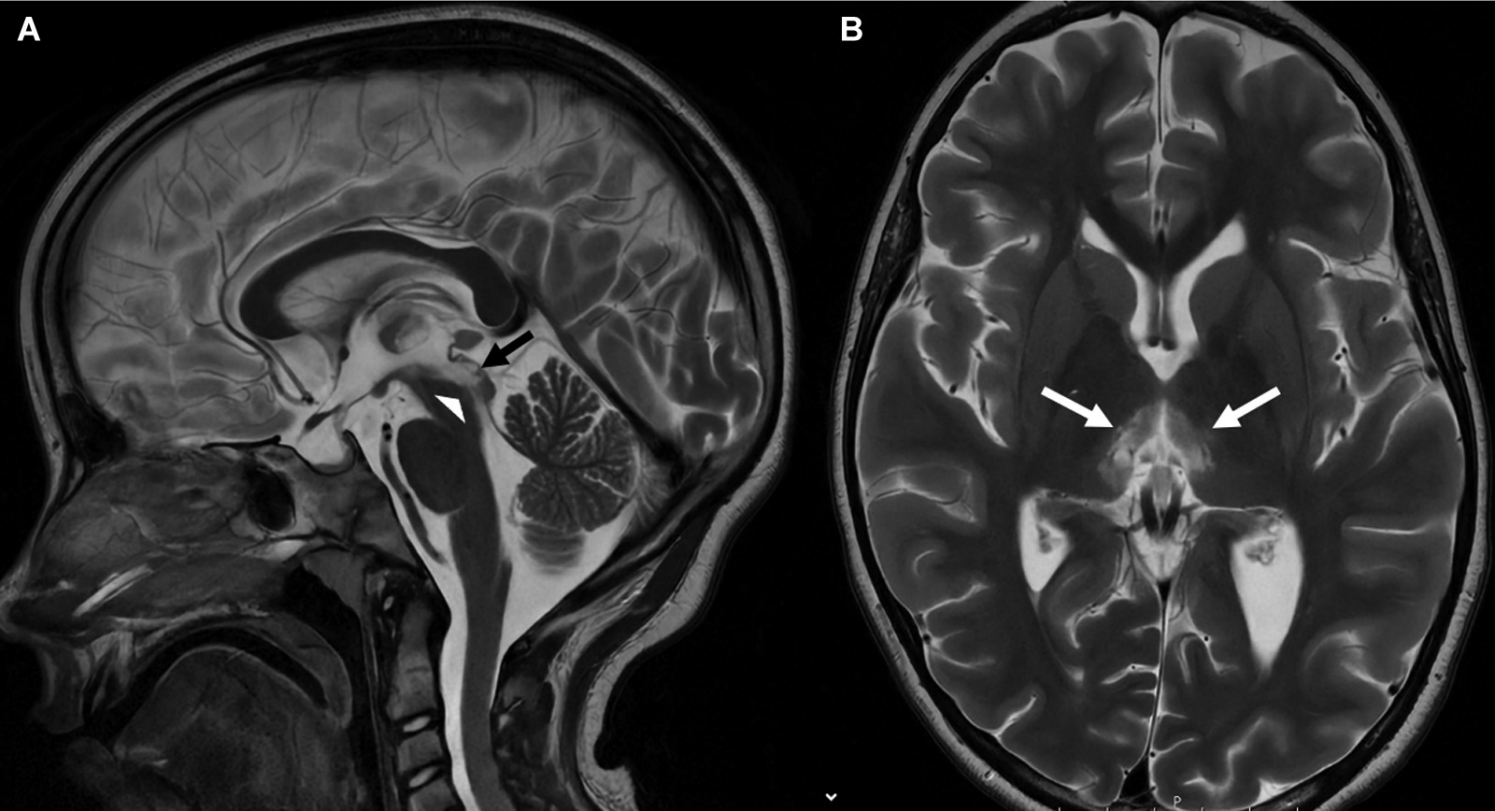
Follow-up magnetic resonance imaging after 3 weeks (T2-weighted sequences sagittal [ **A** ] and transversal [ **B** ]). The lesions show regredient edema, but evolving parenchymal loss in the periaqueductal gray/pretectal area ( *white arrowhead* in **A** ), the superior colliculi ( *black arrow* in **A** ), and the medial thalami ( *white arrows* in **B** ).

Blood analysis showed no sign of inflammation, with decreased thyroid parameters interpreted as low-T3 syndrome due to severe illness. Anti-dsDNA antibodies were undetectable, and antinuclear antibodies were slightly elevated. Following the diagnosis of CSF pleocytosis, immediate treatment was initiated with a 5-day intravenous steroid pulse (methylprednisolone 20 mg/kg), followed by gradual tapering. The clinical status remained unchanged during the following week. Therapy was therefore escalated and immunoadsorption (IA) was administrated five times. Excessive sleep improved significantly; the patient was able to consume increasing amounts of fluids and food orally and was mobilized and able to walk again. However, oculomotor impairment remained, with persistent vertical gaze palsy and absent pupillary response (EDSS 3). Comprehensive opthalmological and neurological examination revealed no further abnormalities. Follow-up cranial MRI after 3 weeks revealed regressing edema but evolving parenchymal defects in the affected areas. No new lesions were found ( Fig. 2 ).

Disease-modifying therapy with an anti-IL6 receptor antibody (tocilizumab) was initiated, and the patient was transferred to a pediatric rehabilitation center.

## Discussion

Neuromyelitis optica spectrum disorder (NMOSD) is an autoimmune inflammatory disease affecting the central nervous system. 1 As the first demyelinating disorder with an identified specific target antigen, 2 NMOSD has been the focus of numerous recent studies. Although it is primarily considered an adult disease, pediatric onset is reported in 5.3% of cases, predominantly in females with a median age of 12 years, 3 4 aligning with our patient's presentation.

Historically, optic neuritis and myelitis were mandatory characteristics for diagnosis, 5 as they were reported in almost every patient. 3 Interestingly, our patient did not exhibit these symptoms. Since 2015, revised diagnostic criteria require at least one of several predefined core characteristics as well as a positive test for AQP4-IgG antibodies. 6 Besides optic neuritis and acute myelitis core clinical characteristics are area postrema syndrome, acute brainstem syndrome, symptomatic narcolepsy, and other cerebral symptoms with NMOSD typical lesions. In our patient, we observed symptomatic narcolepsy, area postrema syndrome, and a central oculomotor disorder with absent pupillary light response related to an acute lesion of the pretectal area, tegmentum mesencephali, and colliculi superiores. NMOSD was suspected and diagnosis was confirmed by detection of AQP4-IgG antibodies in serum. Lesions in the area postrema and periaqueductal gray are typical MRI findings that raise radiological suspicion for NMOSD, 6 whereas bithalamic lesions have wide differentials, including inflammatory, infectious, metabolic, toxic, neoplastic, and vascular etiologies. 7 However, the involvement of the periependymal surface around the third ventricle involving the medial thalami is also a classic finding of NMOSD. 8 Additionally, the absence of OCBs 6 supported the diagnosis.

Acute therapy followed the latest recommendations, including high-dose glucocorticoids followed by a tapering oral steroid regimen over several months. 9 Due to a lack of clinical improvement, IA therapy was added early and five cycles were performed. 9 Both IA and therapeutic plasma exchange are efficient treatment options in NMOSD. 10 We chose IA because it is usually well tolerated and may have a longer therapy response. Although many inflammatory brain lesions decrease and eventually resolve over several months, 11 it is notable that in our patient, the lesions had already progressed into parenchyma loss on follow-up MRI just 3 weeks after the initial acute attack, despite immediate and aggressive treatment.

In NMOSD lifelong disease-modifying therapy is recommended, as only 5 to 10% of patients experience a single attack. 6 AQP4-positive patients even have an even higher relapse rate compared with seronegative patients. 6 Although it is assumed that many children may have a monophasic disease, 12 up to 45% of seropositive children experience multiple relapses, potentially leading to residual disability with each episode. 3

Therapies target three disease mechanisms: B-cells, IL-6, and the complement system. 13 Effectiveness of rituximab, a monoclonal antibody directed against the CD20 protein, has been demonstrated in a retrospective cohort study 14 and is therefore first-line therapy for children under 12 years of age. Satralizumab, a new anti-IL-6 antibody, is considered one of the most promising long-term treatments in adults, significantly reducing relapse risk. 15 However, it is only approved for children over 12 years of age. 16 For our patient we chose an anti-IL-6-antibody (tocilizumab), due to reports on efficacy and favorable tolerance in children. Additionally, tocilizumab has the same mechanism of action as satralizumab and as only a few months were needed to be bridged to switch to satralizumab treatment soon after the patients 12th birthday the switch could be performed without any precautions.

Our case illustrates the diverse symptoms of AQP4-positive NMOSD in a pediatric patient without involvement of the optic nerves or spinal cord. MRI findings in the area postrema, the periaqueductal gray, and other periependymal surface localizations strongly suggest the diagnosis of NMOSD. Therefore, NMOSD should also be considered as a differential diagnosis in children with bithalamic MRI lesions. Permanent sequelae have been reported even after the first attack. Although follow-up is still short in our patient, we do not expect full recovery due to the parenchymal loss already visible on follow-up MRI. Long-term immunotherapy is clearly recommended, targeting three different disease mechanisms, yet data on efficacy and safety are still scarce, especially in children.
